# Miniature CRISPR-Cas12f1-Mediated Single-Nucleotide Microbial Genome Editing Using 3′-Truncated sgRNA

**DOI:** 10.1089/crispr.2022.0071

**Published:** 2023-02-09

**Authors:** Ho Joung Lee, Hyun Ju Kim, Sang Jun Lee

**Affiliations:** Department of Systems Biotechnology, Institute of Microbiomics, Chung-Ang University, Anseong, Republic of Korea.

## Abstract

The CRISPR-Cas system has been used as a convenient tool for genome editing because the nuclease that cuts the target DNA and the guide RNA that recognizes the target are separated into modules. Cas12f1, which has a smaller size than that of other Cas nucleases, is easily loaded into vectors and is emerging as a new genome editing tool. In this study, AsCas12f1 was used to negatively select only *Escherichia coli* cells obtained by oligonucleotide-directed genome editing. Although double-, triple-, and quadruple-base substitutions were accurately and efficiently performed in the genome, the performance of single-base editing was poor. To resolve this limitation, we serially truncated the 3′-end of sgRNAs and determined the maximal truncation required to maintain the target DNA cleavage activity of Cas12f1. Negative selection of single-nucleotide-edited cells was efficiently performed with the maximally 3′-truncated sgRNA–Cas12f1 complex *in vivo*. Moreover, Sanger sequencing showed that the accuracy of single-nucleotide substitution, insertion, and deletion in the microbial genome was improved. These results demonstrated that a truncated sgRNA approach could be widely used for accurate CRISPR-mediated genome editing.

## Introduction

CRISPR-Cas is a microbial adaptive immune system that recognizes and cleaves similar sequences of foreign nucleic acids derived from phages and plasmids, which are “memorized” in the genome of various microbes.^1^ Target nucleic sequences are recognized by base pairing with guide RNAs.^2^ Proteinaceous Cas nucleases can cleave any target DNA sequence by altering the target recognition sequence (TRS) of guide RNAs.^3^ Therefore, CRISPR-Cas technology has broad applications in the genome editing of microorganisms,^4^ animals,^5^ plants,^6^ and humans.^7^

However, the CRISPR-Cas system allows several base pair mismatches between the target DNA and the guide RNA, which can interfere with the accuracy of CRISPR-Cas-mediated genome editing.^8^ Off-target DNA cleavage has been observed in eukaryotic systems with large sized genomes.^9^ For safe on-target editing without DNA cleavage, base editors and prime editors have been developed by fusing base deaminase or reverse transcriptase with deactivated Cas nucleases.^10^ However, the large size of the fused construct is not suitable for loading into a vector with limited size.

More than 90 Cas nucleases, including Cas9 and Cas12a (Cpf1), have been identified thus far^11,12^ and used to develop genome editing tools.^13^ Recently, Cas nucleases with a relatively small size, such as Cas12e (CasX)^14^ and Cas12j (CasΦ),^15^ were reported. Cas12f1, which was discovered in metagenomic data sets,^16^ has attracted much attention due to its small size.

Studies have been conducted on the crystal structure of the Cas12f1–sgRNA complex^17,18^ and sgRNA engineering^19,20^ for genome editing tool development. Un1Cas12f1 derived from uncultured archaea has been used for indel formation,^19^ base editing,^20^ and gene regulation^21^ in human cells, as well as 1 kb-level gene deletion in *Escherichia coli.*^22^ The smallest Cas12f1 ortholog AsCas12f1 derived from *Acidibacillus sulfuroxidans*^23^ has been used for 1 kb-level gene deletion in *Bacillus anthracis.*^24^

In microbial genome editing, the CRISPR-Cas system recognizes and cuts unedited target DNA, leaving the edited sequence created by oligonucleotide-directed mutagenesis, which is known as negative selection.^25^ Cas nucleases have a mismatch tolerance, which allows them to cut the target DNA even if there is mispairing between the target DNA and the guide RNA.^26^ Therefore, single-base genome editing has rarely been possible.^27^ In contrast, the target-mismatched guide RNA method allows precise single-base editing using Cas9^8^ and Cas12a.^28^ In addition, PAM-distal truncation in guide RNAs allows single-nucleotide substitution and indel in various CRISPR-Cas systems including Cas9-NG^29^ and Cas12a.^27^

Cas12f1 shows several characteristics similar to those of the existing Cas12 nuclease as follows: it has a T-rich PAM sequence, the RuvC domain is involved in target DNA cleavage, and the protein of the effector complex consists of a single polypeptide.^18^ However, Cas12f1 has characteristics that distinguish it from other Class II Cas nucleases. Cas12f1 actually functions as a multiprotein complex by forming a dimer,^18^ and its unique features include inconsistent DNA cleavage sites.^23^ In this study, we used the truncated guide RNA method to perform single-nucleotide genome editing with a smaller dimerized Cas12f1 nuclease.

## Materials and Methods

### Strains and culture conditions

The *E. coli* strains used are listed in Supplementary Table S1. They were grown in LB broth at 30°C or 37°C depending on the *ori* sequence of the plasmids. *E. coli* DH5α was used for the construction and cloning of sgRNA plasmids. *E. coli* MG1655 was used for the genomic integration of the *Ascas12f1* gene. When needed, ampicillin, kanamycin, and spectinomycin were added to the medium at 50, 25, and 75 μg/mL, respectively. To obtain electrocompetent cells, the *E. coli* cells were cultured in LB broth at 30°C until the optical density at 600 nm reached 0.4.

Subsequently, the cells were harvested, washed, and resuspended in 10% glycerol solution. Finally, they were aliquoted and stored at −80°C. For the overexpression of λ-red recombinases and AsCas12f1 protein, l-arabinose (final 1 mM) was added when the optical density at 600 nm (OD_600_) approached 0.4. Then, the cells were cultured for another 3 h.

### Genomic integration

The primers used for the DNA amplification of *E. coli* strains are listed in Supplementary Table S2. For the integration of the *Ascas12f1* gene in the chromosomal DNA of *E. coli*, *Ascas12f1* was PCR amplified using the plasmid p15a-AsCas12f-apmR (p15a-AsCas12f-apmR was a gift from Quanjiang Ji, Addgene plasmid #171610; http://n2t.net/addgene:171610; RRID: Addgene_171610) and fused with the P_BAD_ construct and a kanamycin resistance marker through splice-overlap PCR to generate an *Ascas12f1*–KmR cassette. Purified PCR products were electroporated into λ-red recombinases overexpressing *E. coli* MG1655 for the genomic integration of the *cas9* gene in the arabinose operon. The strain was designated as *E. coli* HL061.

### Plasmid construction

Supplementary Table S1 lists the sgRNA plasmids used for the single-nucleotide editing of *galK*, and the primers used are listed in Supplementary Table S2. sgRNA expression plasmid vectors were constructed by targeting the *galK* of *E. coli* (497–516th bases) for single-base genome editing. The sgRNA sequence was amplified from the plasmid psgRNAv1 empty (psgRNAv1 empty was a gift from Quanjiang Ji, Addgene plasmid #171611; http://n2t.net/addgene:171611; RRID: Addgene_171611) and ligated with the fragment from pHK459^30^ containing a spectinomycin resistance gene and pJ23119 for sgRNA expression through Gibson assembly.

Finally, pHL267 plasmid targeting *galK* gene was constructed. All other sgRNA plasmids including sgRNA plasmids targeting *xylB* gene were constructed by Gibson assembly of two amplified DNA fragments using pHL267 as a template.

### Genome editing

For oligonucleotide-directed nucleotide editing in the *galK* or *xylB* gene, stop codon-encoding mutagenic oligonucleotides (100 pmol) were electroporated with sgRNA plasmids (200 ng) into HL061 cells where both λ-red Bet and AsCas12f1 were overexpressed as a result of l-arabinose induction. The mutagenic oligonucleotides used for genome editing are listed in Supplementary Table S3. Electroporation was performed using a 0.1 cm electroporation cuvette at 25 μF, 200 Ω, and 1.8 kV. The electroporated cells were immediately transferred to 950 μL of SOC and recovered for 1 h at 37°C and 180 rpm.

Subsequently, they were spread on MacConkey agar containing d-galactose (0.5%, CAS No. 59-23-4) or d-xylose (0.5%, CAS No. 58-86-6), and incubated for 16 h at 37°C. The percentage of white colonies to total colonies was calculated to estimate the single-nucleotide editing efficiency. Randomly selected white colonies were Sanger sequenced to assess the editing accuracy of AsCas12f1.

## Results

### Oligonucleotide-directed genome editing using CRISPR–AsCas12f1

**FIG. 1.**
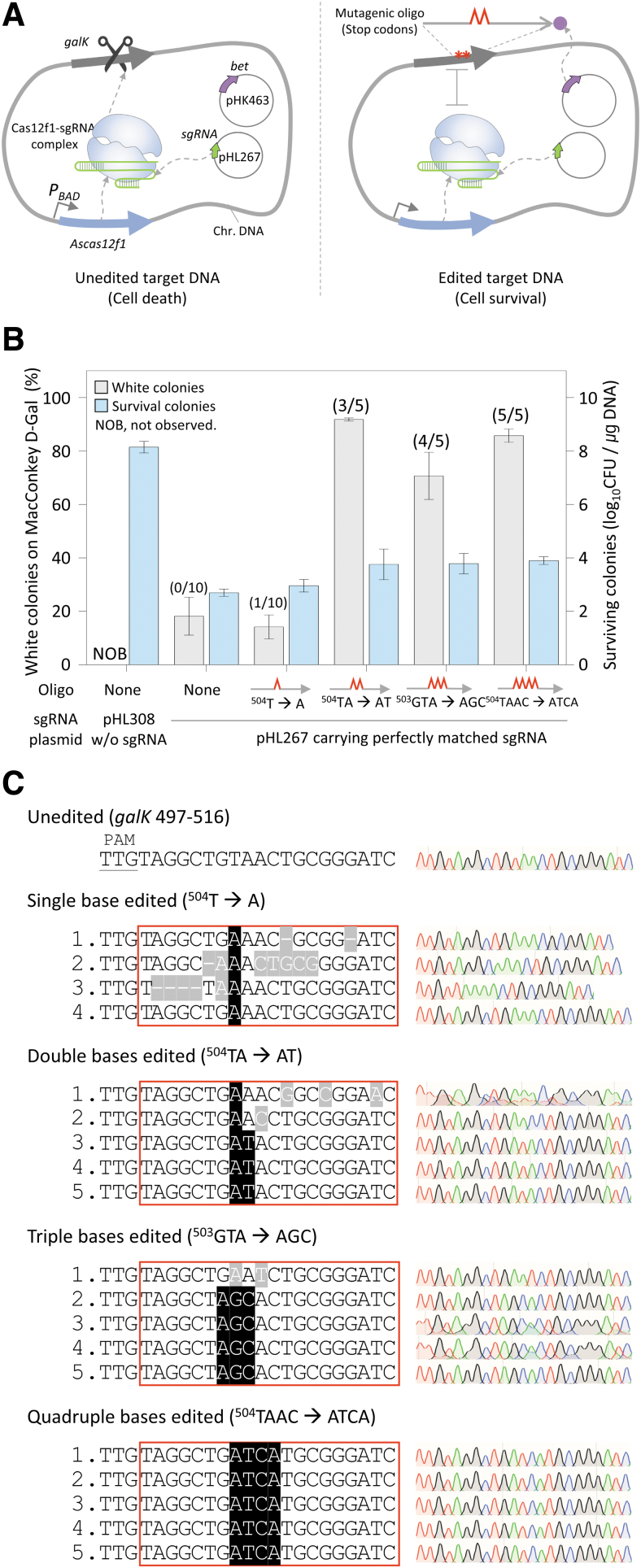
Oligonucleotide-directed microbial genome editing by CRISPR–AsCas12f1. **(A)** Negative selection of genome-edited cells by the sgRNA–AsCas12f1 system. The AsCas12f1 nuclease expressed by the genome and sgRNA expressed by the plasmid form AsCas12f1–sgRNA complex, which cleaves the unedited *galK* target DNA and causes cell death (left). When the *galK* gene is mutated by mutagenic oligonucleotides, it is not recognized as a target, and the cell survives (right). Red cones and asterisks indicate mismatched bases and DNA mutations, respectively. **(B)** Efficiency and accuracy of CRISPR–AsCas12f1-mediated microbial nucleotide editing. Efficiency (%) was calculated as the number of white colonies of the total red and white colonies. Accuracy was calculated as the number of correctly edited colonies among selected white colonies confirmed by Sanger sequencing (indicated in the parentheses). The number of surviving colonies indicates the success of negative selection, which shows whether the CRISPR–AsCas12f1 complex could properly cleave the target DNA and cause cell death. Each bar represents the mean of three independent experiments. **(C)** Sanger sequences of single or multiple base-edited *galK* targets in cells showing white colonies on MacConkey agar containing d-galactose with sgRNA–Cas12f1 negative selection. Red boxes indicate the complementary region to the sgRNA. Black- and gray-shaded letters indicate correctly edited bases and unwanted mutations, respectively.

To determine the efficiency of oligonucleotide-directed microbial genome editing when the AsCas12f1–sgRNA complex was used for negative selection, sgRNA plasmids and mutagenic oligonucleotides that could induce the mutations of one to four bases were electroporated into cells expressing AsCas12f1 and λ Bet. The AsCas12f1–sgRNA complex recognizes and cleaves a DNA target that does not have the intended mutation, causing cell death. In contrast, a mutated target is not recognized by the AsCas12f1–sgRNA complex, and the cell survives, which is known as negative selection (Fig. 1A).

The transformation efficiency of the sgRNA-deleted plasmid (pHL308) into cells expressing AsCas12f1 and λ Bet was 10^8.1^ CFU/μg DNA. However, when the plasmid expressing sgRNA (pHL267) was introduced, the transformation efficiency was reduced to 10^2.7–3.9^ CFU/μg DNA (Fig. 1B). The markedly lower number of surviving colonies after transformation indicated that the AsCas12f1–sgRNA complex could properly recognize and cut the target dsDNA in the genome, causing cell death.

Oligonucleotides carry stop codons in the target *galK* gene, causing the premature translational termination of GalK proteins. Edited cells form white colonies on MacConkey agar containing d-galactose, and editing efficiency can be easily confirmed by phenotypic changes.^29^ When two, three, and four bases were edited, the percentages of white colonies of the total colonies were 85%, 70%, and 80%, respectively (Fig. 1B), and Sanger sequencing confirmed that 3, 4, and 5 of the 5 white colonies were altered correctly, respectively (Fig. 1C).

However, in single-base editing, the percentage of white colonies was significantly reduced to 12% (compared with the editing of two or more bases), and only 4 of 10 colonies were successfully amplified by PCR. Sanger sequencing revealed that only one of the four colonies had correctly edited nucleotides. Unexpectedly, even when mutagenic oligonucleotides were not added, the white colony percentage was ∼18%. PCR was attempted on 10 white colonies; however, none of them were amplified. The DNA deletion at the site cleaved by the Cas12f1–sgRNA complex appeared to be outside the primer range. These results demonstrated that the efficiency and accuracy of editing two or more bases using AsCas12f1 were high. However, both efficiency and accuracy in single-base editing were rather low.

### Accurate single-base editing in the *galK* gene by 3′-truncated sgRNA–AsCas12f1

**FIG. 2.**
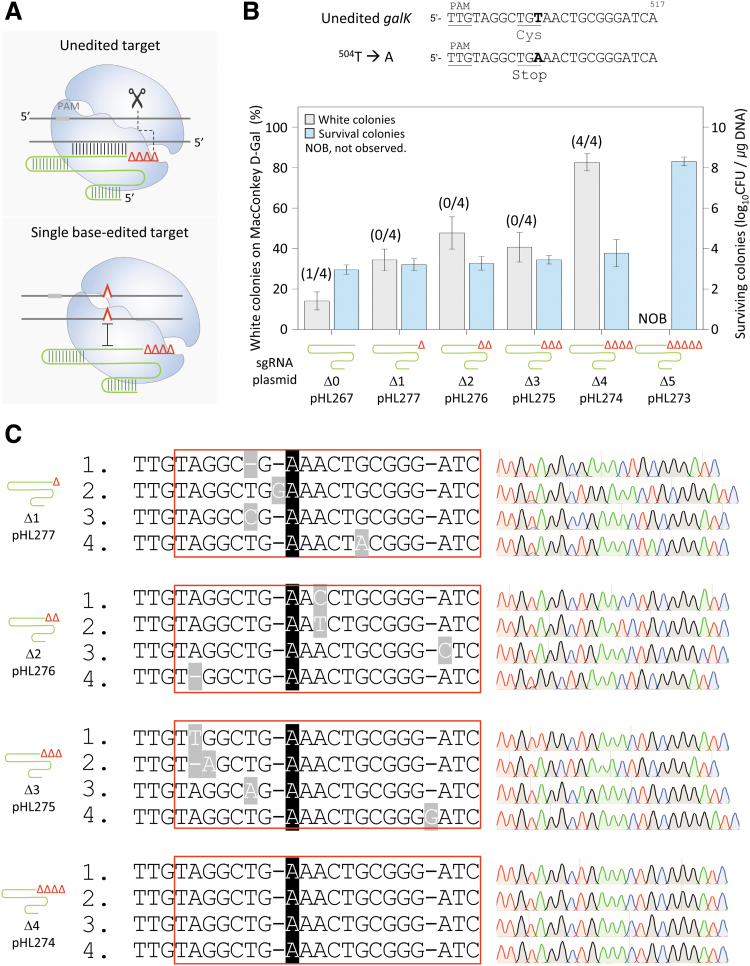
Single-base editing by the maximally 3′-truncated sgRNA–AsCas12f1 complex. **(A)** Mismatch intolerance of CRISPR–AsCas12f1. The maximally 3′-truncated sgRNA–AsCas12f1 complex recognizes and cleaves an unedited target (top) and does not recognize a single-base-edited target (bottom). **(B)** Single-base editing using 3′-truncated sgRNAs. Δ1 to Δ5 represent the number of truncated nucleotides at the 3′-end of the sgRNA. Genome editing efficiency was calculated as the proportion of white colonies formed on MacConkey agar containing d-galactose. Numbers in parentheses are correctly edited colonies confirmed by Sanger sequencing/white colonies selected for sequence analysis. The number of surviving colonies was calculated to determine whether negative selection was successful based on the target cleavage activity of the Cas12f1–sgRNA complex. The red Δ represents a nucleotide truncation at the 3′-end of the sgRNA. Each bar represents the mean of three independent experiments. **(C)** Sanger sequences of single-base-edited *galK* targets in cells showing white colonies on MacConkey agar containing d-galactose with 3′-truncated sgRNA–Cas12f1 negative selection. Red boxes indicate the complementary region to the sgRNA. Black- and gray-shaded letters indicate correctly edited bases and unwanted mutations, respectively.

We attempted to determine the length of the TRS of the sgRNA required to recognize a target and maintain the DNA cleavage activity of the AsCas12f1–sgRNA complex (Fig. 2A). The minimum sgRNA length capable of recognizing a target DNA sequence was regarded as ideal for the negative selection of single-nucleotide-edited targets considering that a single mismatch between the target DNA and sgRNA can prevent target recognition. When one to four nucleotides were truncated at the 3′-end of sgRNAs, the number of surviving colonies was similar at around 10^2.7–3.6^ CFU/μg DNA.

However, when five nucleotides were truncated at the 3′-end, the number of surviving colonies was increased to 10^8.1^ CFU/μg DNA, which was similar to the number of transformants when sgRNA-deleted plasmids were electroporated (Supplementary Fig. S1).

Based on the mentioned results, the AsCas12f1-mediated single-nucleotide editing efficiency was compared using sgRNAs with one to five nucleotides truncated at the 3′-end (Fig. 2B). When the number of truncated nucleotides at the 3′-end of the sgRNA was one to three, the proportion of white colonies was 34–47%. However, when the four nucleotide-truncated sgRNA was used, the percentage of white colonies was markedly increased to 82%. When the five nucleotide-truncated sgRNA was used, no white colony was formed, and the CFU was significantly increased to 10^8.3^ CFU/μg DNA, indicating the failure of negative selection.

The accuracy of single-nucleotide editing was analyzed by Sanger sequencing. When three nucleotides or less were truncated at the 3′-end of sgRNAs, nucleotide sequence changes other than the intended *galK* T504A mutation were also observed. In contrast, in the case of the truncation of four nucleotides, which was considered as using a maximally 3′-truncated sgRNA for target cleavage, we observed correct single-nucleotide editing (T504A) among all of the four white colonies selected for sequence analysis (Fig. 2C). These results demonstrated that the editing efficiency and accuracy of AsCas12f1 were significantly improved using a 3′-truncated sgRNA.

### Single-nucleotide indel in the *galK* gene using 3′-truncated sgRNA

**FIG. 3.**
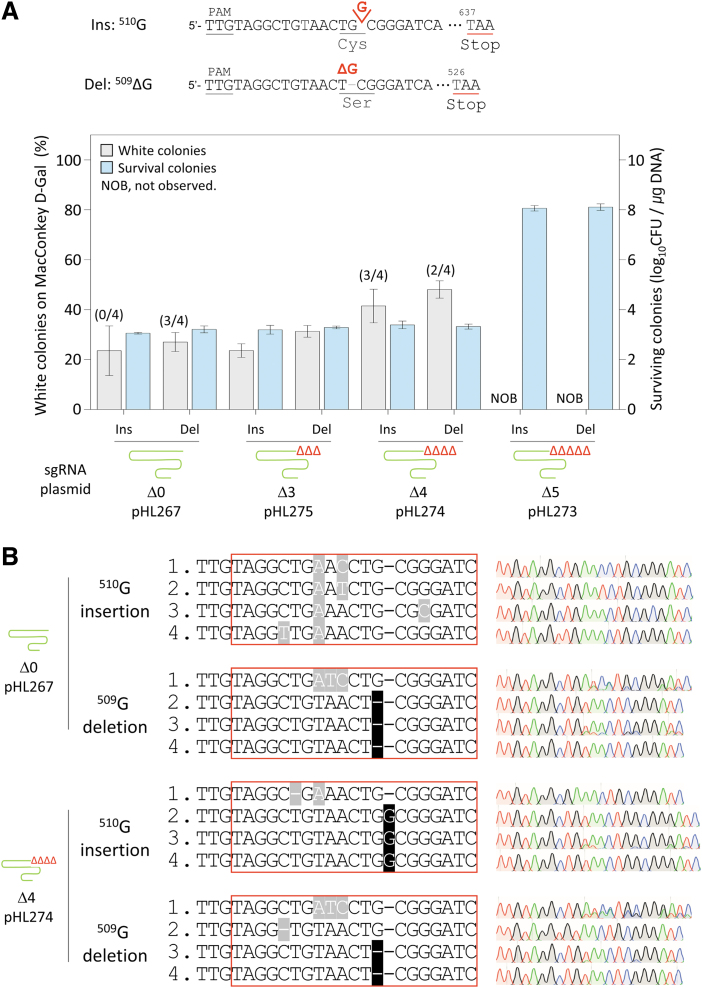
Genomic insertion and deletion of single nucleotides with 3′-truncated sgRNA–AsCas12f1. **(A)** Efficiency of phenotypic changes and number of surviving colonies during single-nucleotide insertion and deletion in the *galK* gene. Δ0, Δ3, Δ4, and Δ5 represent the number of truncated nucleotides at the 3′-end of the sgRNA. Editing efficiency was calculated as the proportion of white colonies formed on MacConkey d-galactose agar due to the *galK* mutation among all colonies. The number of surviving colonies was calculated to confirm the target cleavage activity of the sgRNA–Cas12f1 complex. Each bar represents the mean of three independent experiments. **(B)** Sanger sequences of single-nucleotide-edited *galK* targets with untruncated or maximally truncated sgRNA–AsCas12f1. Red boxes indicate the complementary region to the sgRNA. Black- and gray-shaded letters indicate correctly edited bases and unwanted mutations, respectively.

We investigated whether 3′-truncated sgRNA–AsCas12f1 could efficiently edit the insertions and deletions of single nucleotides in the genome. When the nucleotide G^509^ of the *galK* gene was deleted or when G was inserted at position 510, the frameshift caused the premature translation termination of GalK proteins by generating stop codons at positions 526 and 637 (Fig. 3A). As a result, when there was no 3′-truncation in the sgRNA, the percentage of white colonies with G^510^ insertion was 23%, and the percentage of white colonies with G^509^ deletion was 27%.

When three nucleotides were deleted from the sgRNA, the percentage of white colonies with G^510^ insertion was 23%, and the percentage of white colonies with G^509^ deletion was 31%, which were similar to the results for the 3′-untruncated sgRNA. When four nucleotides were truncated at the 3′-end of the sgRNA, the percentages of white colonies with G^510^ insertion and G^509^ deletion were increased to 41% and 48%, respectively. When five nucleotides were truncated at the 3′-end of the sgRNA, the number of surviving colonies was markedly increased to 10^8.1^/μg DNA, and white colonies were not observed.

Sanger sequencing was performed to confirm the editing accuracy (Fig. 3B). When the 3′-untruncated sgRNA was used, in the case of G^509^ deletion, three of four white colonies showed accurate editing; however, in the case of G^510^ insertion, peripheral sequence changes were observed in all four colonies. When the sgRNA with 3′-truncation of four nucleotides was used, two of four white colonies in the case of G^509^ deletion and three of four white colonies in the case of G^510^ insertion showed accurate editing results.

### Single-nucleotide editing in the *xylB* gene using 3′-truncated sgRNA

**FIG. 4.**
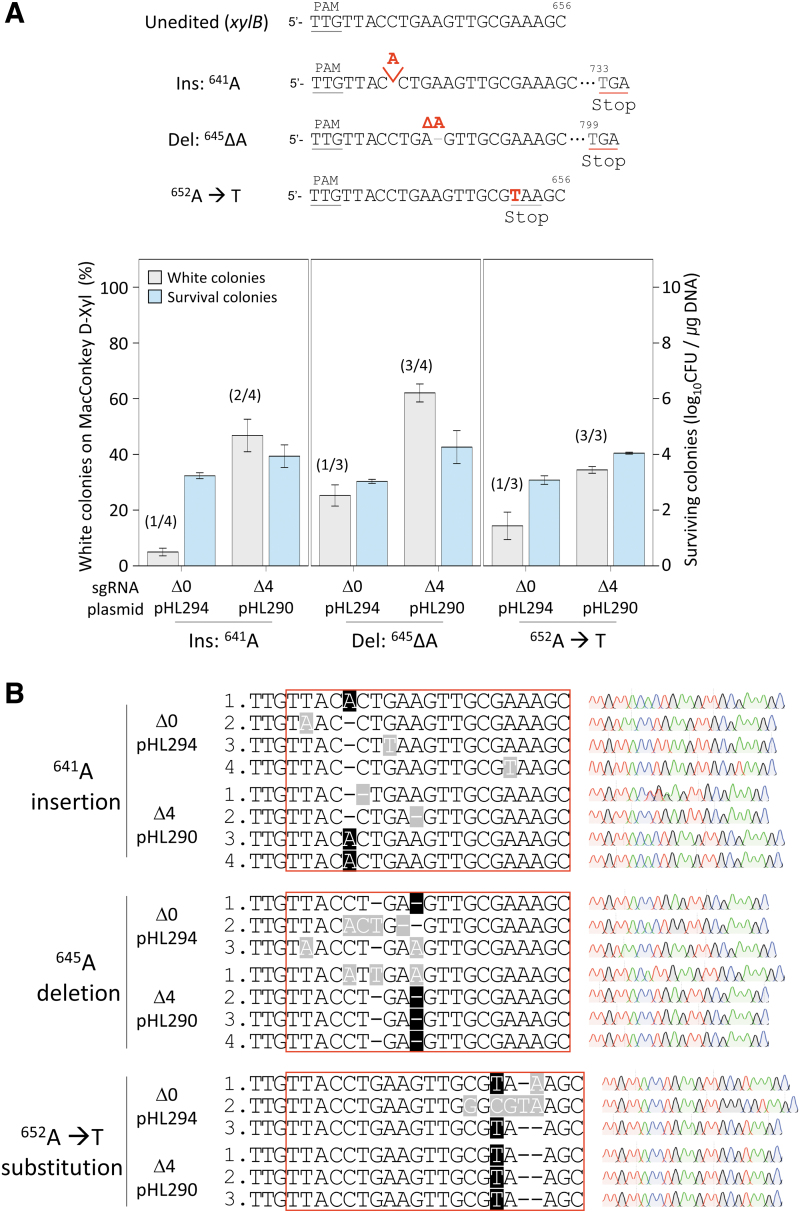
Insertion, deletion, and substitution of single nucleotides in the *xylB* gene with 3′-truncated sgRNA–AsCas12f1. **(A)** Efficiency of phenotypic changes and number of surviving colonies during single-nucleotide insertion, deletion, and substitution in the *xylB* gene. Δ0 and Δ4 represent the number of truncated nucleotides at the 3′-end of the sgRNA. Editing efficiency was calculated as the proportion of white colonies formed on MacConkey d-xylose agar due to the *xylB* mutation among all colonies. The number of surviving colonies was calculated to confirm the target cleavage activity of the sgRNA–Cas12f1 complex. Each bar represents the mean of three independent experiments. **(B)** Sanger sequences of single-nucleotide-edited *xylB* targets with untruncated or maximally truncated sgRNA–AsCas12f1. Red boxes indicate the complementary region to the sgRNA. Black- and gray-shaded letters indicate correctly edited bases and unwanted mutations, respectively.

To examine the effect of using the 3′-truncated sgRNA on editing efficiency and accuracy with an additional target, we chose the 641st, 645th, and 652nd nucleotides in the *xylB* gene for insertion, deletion, and substitution, respectively. Mutagenic oligonucleotides generate stop codons in the *xylB* gene target, causing the premature translational termination of XylB proteins. Edited cells form white colonies on MacConkey agar containing d-xylose, and editing efficiency can be confirmed by phenotypic changes. The sgRNA with 3′-truncation of four nucleotides was more effective than the 3′-untruncated sgRNA in all the types of editing (Fig. 4A). The accuracy of single-nucleotide editing in the *xylB* gene was also analyzed by Sanger sequencing.

When the 3′-untruncated sgRNA was used, only one of four white colonies in the case of A^641^ insertion and one of three white colonies in the case of A^645^ deletion and ^652^A to T substitution showed accurate editing. However, when the sgRNA with 3′-truncation of four nucleotides was used, two of four white colonies in the case of A^641^ insertion and three of four white colonies in the case of A^645^ deletion showed accurate editing results. We observed correct single-nucleotide substitution (A652T) among all of the three white colonies selected for sequence analysis (Fig. 4B). These results demonstrated that the use of the 3′-truncated sgRNA increased the editing efficiency and accuracy for insertion, deletion, and substitution at the single-nucleotide level.

## Discussion

The characteristics of cells are dependent on the information stored in their genomic DNA sequence, and the nucleotide sequence of the genome continues to change dynamically through adaptation and evolution. A single-nucleotide mutation in a certain gene may alter the strength of the promoter and amino acids in the open reading frame, which can change the function of the gene,^31^ causing it to lose its original function^32^ or acquire a new function.^33^ Over the past decade, the CRISPR-Cas system has evolved into a single-nucleotide editing tool for restoring function to cells, conferring new functions, or enhancing cell performance.^34,35^

Reportedly, Cas12f1 rarely tolerates a mismatched base pair between the TRS of the sgRNA and the target DNA sequence.^19,24^ Therefore, it was anticipated that AsCas12f1, which has a small size, may be effective for the negative selection of single-nucleotide-edited cells. However, we observed that the single-nucleotide editing of AsCas12f1 was not efficient compared with double-, triple-, and quadruple-base editing (Fig. 1). The efficiency of single-base substitution (phenotypic changes of white colonies) was 14%, and accurate single-nucleotide editing was observed in only one white colony among four randomly selected white colonies. These results were similar to those for Cas9^8^ and Cas12a,^28^ where a single-base substitution was introduced in the genome with the negative selection of edited cells.

In our previous genome-editing experiments with Cas9 and Cas12a, white colonies were not formed on MacConkey agar containing d-galactose in the absence of mutagenic oligonucleotides.^8,27,29^ However, in the case of AsCas12f1, white colonies were formed at a significant level even without mutagenic oligonucleotides. Analysis of the target DNA sequence of one of the white colonies revealed that a substantial length of DNA fragment (∼4.4 kb) including a part of the *gal* operon was deleted (Supplementary Fig. S2).

Several microorganisms including *E. coli* rely on homology-directed recombination rather than nonhomologous end joining as a genome repair method during double-strand breaks.^36^ In the case of Cas12f1, the cause for the formation of white colonies in the absence of mutagenic oligonucleotides is unclear, and further studies should be conducted.

Similar to Cas9 and Cas12a in previous studies,^27,29^ the Cas12f1 nuclease was able to distinguish between single-nucleotide-edited and nonedited targets with the maximally truncated sgRNA, thus allowing single-nucleotide genome editing (Fig. 2). In the base substitution experiment, the percentage of white colonies was increased to 80% with the 3′-truncation of four nucleotides in the sgRNA, and the number of surviving cells was not affected. It is possible that during mutagenesis, Cas12f1 cleaves the target, and the target is repaired repeatedly. Sequencing of white colonies showed that highly accurate nucleotide editing was possible with the 3′-truncation of four nucleotides in the sgRNA.

Unlike base substitution, it is impossible to insert or delete desired nucleotides in the target DNA without the cleavage of phosphodiester bonds. Therefore, the use of Cas nucleases is crucial for indel mutagenesis in the genome. In single-base substitution, the proportion of white colonies was markedly increased with 3′-truncated sgRNAs (Fig. 2B).

However, in the case of indel, the proportion of white colonies was relatively smaller (Fig. 3A), indicating that the efficiency of indel was lower than that of substitution; this may be attributed to differences in the repair mechanism and/or repair efficiency for bubbles and bulges caused by base mismatch and indel, respectively. However, even when white colonies were randomly selected and sequenced after single-nucleotide indel editing, the truncated sgRNA approach showed accurate editing results for both the *galK* and *xylB* targets (Figs. 3B and 4B).

## Conclusions

Our study showed that maximally truncated sgRNAs may be used for the negative selection of cells accurately edited by miniature CRISPR–AsCas12f1 during single-nucleotide genome editing. The study not only suggests a direction for the further development of Cas12f1 editing technology but also contributes to broadening the range of CRISPR-Cas systems that can be selected according to the gene delivery method. This truncated sgRNA approach may be utilized in various fields of biotechnology to facilitate the development of CRISPR-Cas genome editing tools.

## Supplementary Material

Supplemental data

Supplemental data

Supplemental data

Supplemental data

Supplemental data
