# Supplementary material for: Miniature CRISPR-Cas12f1-Mediated Single-Nucleotide Microbial Genome Editing Using 3′-Truncated sgRNA
Source: CRISPR J. 2023 Feb 9;6(1):52–61. doi: 10.1089/crispr.2022.0071 (PMC9942177; doi:10.1089/crispr.2022.0071)
Supplement: Supplemental data [file Suppl_FigS1.pdf]

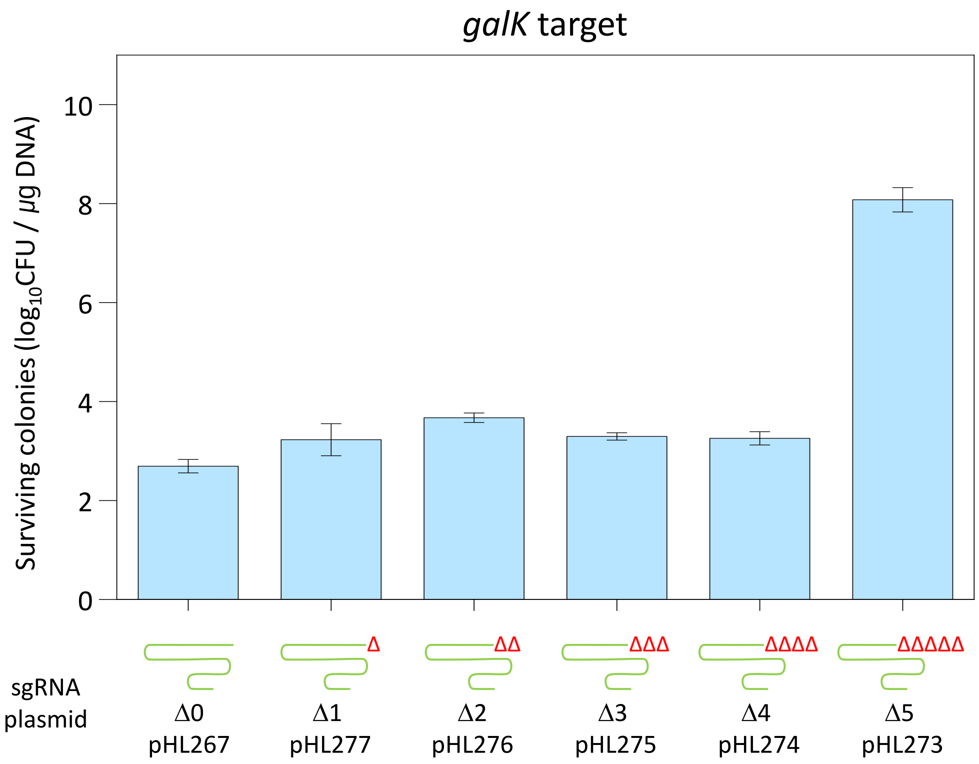

Supplementary Figure S1. 3′-truncated sgRNA plasmids were electroporated into *E. coli* MG1655 cells overexpressing the Cas12f1 protein. The number of surviving colonies for each crRNA plasmid indicates the genomic DNA cleavage activity of the sgRNA/Cas12f1 complex.
