# Supplementary material for: Miniature CRISPR-Cas12f1-Mediated Single-Nucleotide Microbial Genome Editing Using 3′-Truncated sgRNA
Source: CRISPR J. 2023 Feb 9;6(1):52–61. doi: 10.1089/crispr.2022.0071 (PMC9942177; doi:10.1089/crispr.2022.0071)
Supplement: Supplemental data [file Suppl_FigS2.pdf]

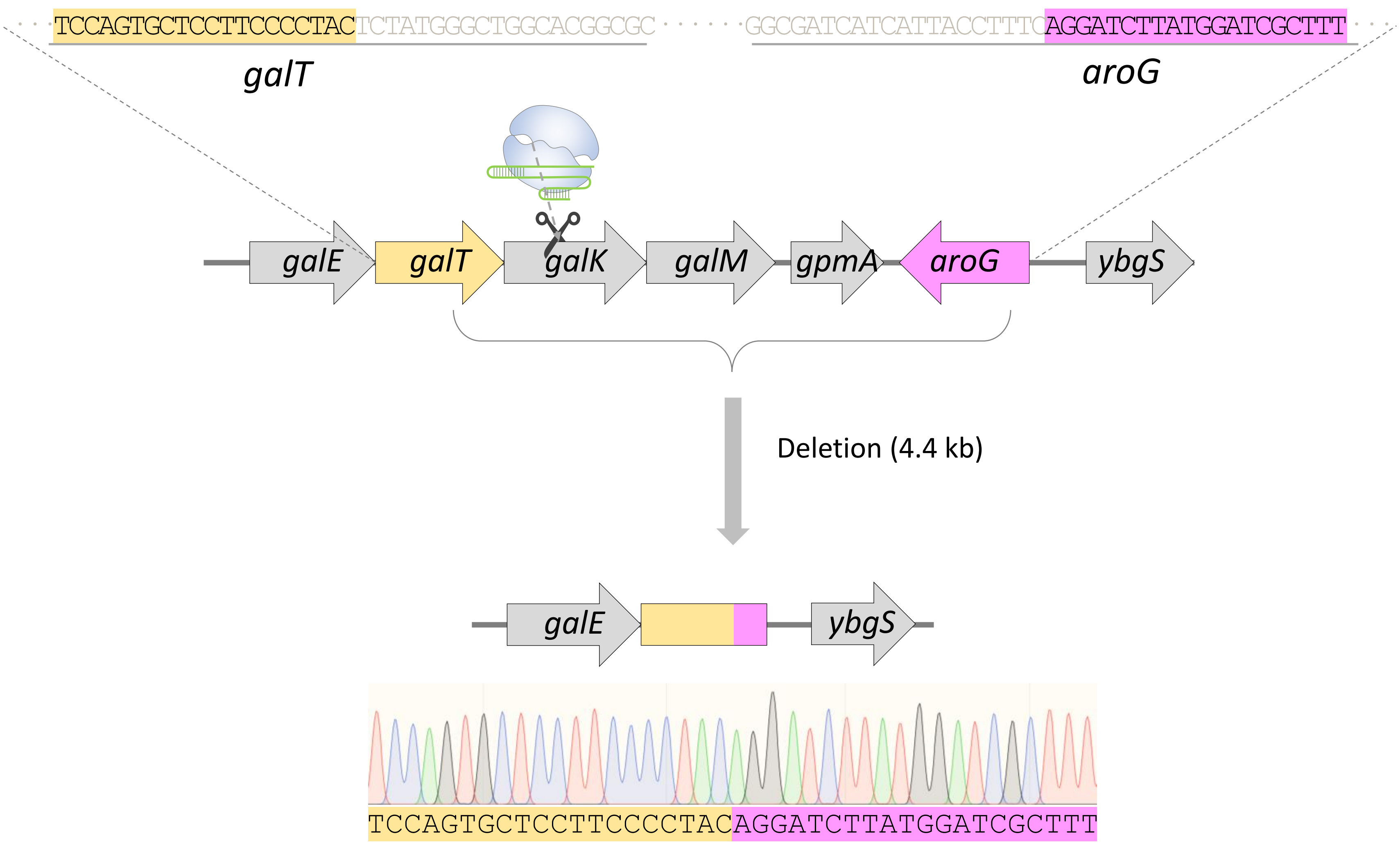

Supplementary Figure S2. Deletion of a large DNA fragment (4.4 kb) including genomic DNA target in the *galK* gene confirmed by Sanger sequencing.
